# Supplementary material for: A simple method to isolate fatty acids and fatty alcohols from wax esters in a wax-ester rich marine oil
Source: PLoS One. 2023 May 12;18(5):e0285751. doi: 10.1371/journal.pone.0285751 (PMC10180661; doi:10.1371/journal.pone.0285751)
Supplement: S1 Table — (PDF) [file pone.0285751.s001.pdf]

Supporting table 1

Weight and percentage of Calanus oil weight of the extracted lipid classes from the 3 individual extraction rounds

	**Extraction 1**		**Extraction 1**		**Extraction 1**	
**Lipid class**	**Weight (mg)**	**% Calanus oil**	**Weight (mg)**	**% Calanus oil**	**Weight (mg)**	**% Calanus oil**
Calanus oil	300	100	336	100	329	100
Neutral lipids	266	89	279	83	274	83
Wax esters	239	80	258	77	255	78
Hydrolyzed wax esters	152	51	164	49	144	44
Free fatty alcohols	81	27	67	20	78	24
Free fatty acids	69	23	56	17	63	19
