# Supplementary material for: A simple method to isolate fatty acids and fatty alcohols from wax esters in a wax-ester rich marine oil
Source: PLoS One. 2023 May 12;18(5):e0285751. doi: 10.1371/journal.pone.0285751 (PMC10180661; doi:10.1371/journal.pone.0285751)
Supplement: S1 File — (PDF) [file pone.0285751.s002.pdf]

Supporting figures

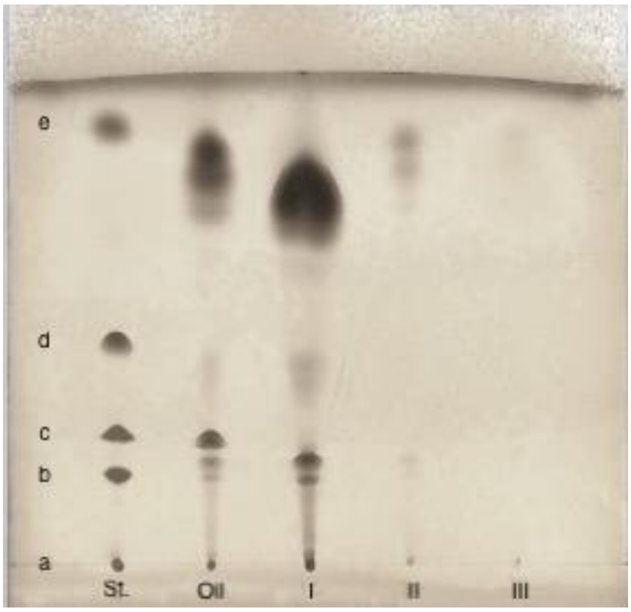

Fig 1: TLC of neutral lipids separated by SPE from 300mg Calanus oil. St.: fatty acid standard 18-5 A (Nu-Chek Prep, INC. USA), containing lecithin (a); cholesterol (b); oleic acid (c); TAG (d); and cholesteryl oleate (e). Oil: oil from *C. finmarchicus*. Lane I: Neutral lipids (NL) eluted with 10 mL chloroform/isopropanol (2:1 v/v), Lane II: NL eluted with an additional 10 mL (20 mL in total), Lane III: NL eluted with a final additional 10 mL (30 mL in total).

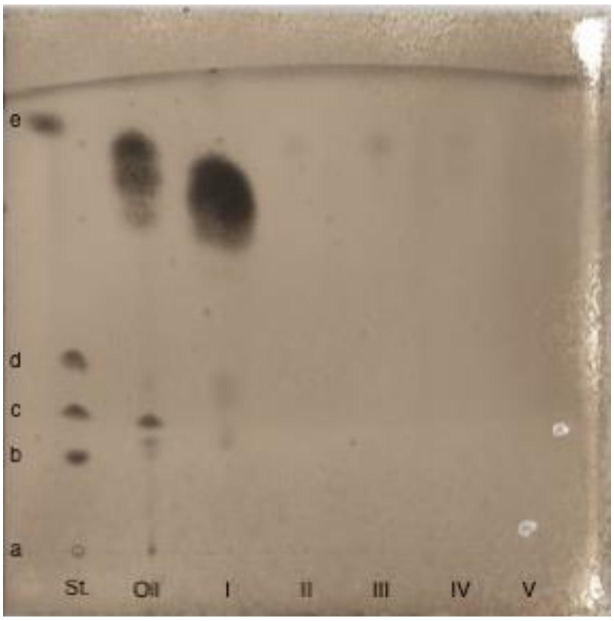

Fig 2: TLC of wax esters separated by SPE from the neutral lipids from 300mg Calanus oil: St.: fatty acid standard 18-5 A (Nu-Chek Prep, INC. USA) containing lecithin (a); cholesterol (b); oleic acid (c); TAG (d); and cholesteryl oleate (e). Oil: oil from *C. finmarchicus*. Lane I: wax esters eluted with 10 mL heptane, Lane II: wax esters eluted with an additional 10 mL (20 mL in total), Lane III: wax esters eluted with an additional 10 mL (30 mL in total), Lane IV: wax esters eluted with an additional 10 mL (40 mL in total), Lane V: wax esters eluted with a final additional 10 mL heptane (50 mL in total).

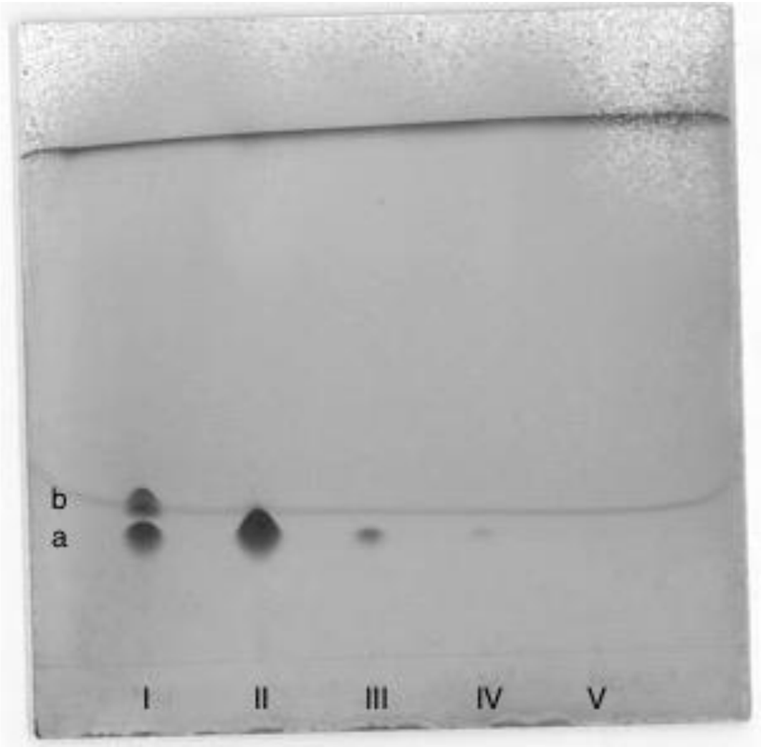

Fig 3: TLC of separated FAOH from the hydrolyzed wax esters originating from 300mg Calanus oil. Lane I: hydrolyzed wax esters from calanus oil with (a) representing the free fatty alcohols, and (b) representing the free fatty alcohols , Lane II: free fatty alcohols eluted from the hydrolyzed wax esters with 10 mL chloroform/isopropanol (2:1 v/v), lane III: free fatty alcohols eluted with an additional 10 mL (20 ml in total), lane IV: free fatty alcohols eluted with an additional 10 mL (30 ml in total), lane V: free fatty alcohols eluted with a final additional 10 mL chloroform/isopropanol (2:1 v/v) (40 ml in total).

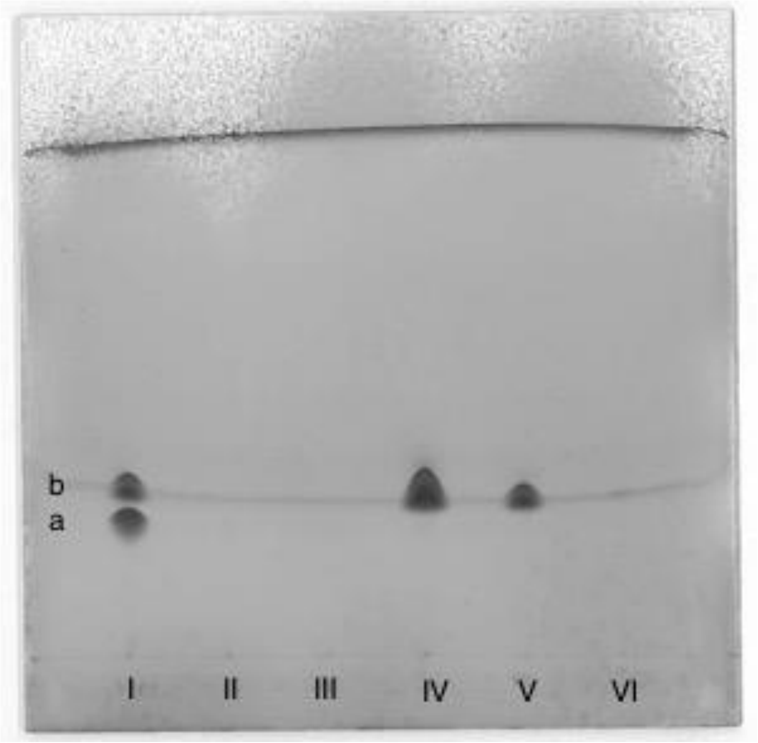

Fig 4: TLC of separated FFA from the hydrolysed wax esters originating from 300mg Calanus oil. Lane I: hydrolyzed wax esters from calanus oil with (a) representing the free fatty alcohols, and (b) representing the free fatty alcohols, Lane II: free fatty acids eluted from the hydrolyzed wax esters with 10 mL diethyl ether/acetic acid (98:2 v/v), Lane III: free fatty acids eluted with an additional 10 mL (20 mL in total), Lane IV: free fatty acids eluted with an additional 10 mL (30 mL in total), Lane V: free fatty acids eluted with an additional 10 mL (40 mL in total), Lane VI: free fatty acids eluted with a final additional 10 mL diethyl ether/acetic acid (98:2 v/v) (50 mL in total).
