# Supplementary material for: A simple method to isolate fatty acids and fatty alcohols from wax esters in a wax-ester rich marine oil
Source: PLoS One. 2023 May 12;18(5):e0285751. doi: 10.1371/journal.pone.0285751 (PMC10180661; doi:10.1371/journal.pone.0285751)
Supplement: S1 Raw images — (PDF) [file pone.0285751.s003.pdf]

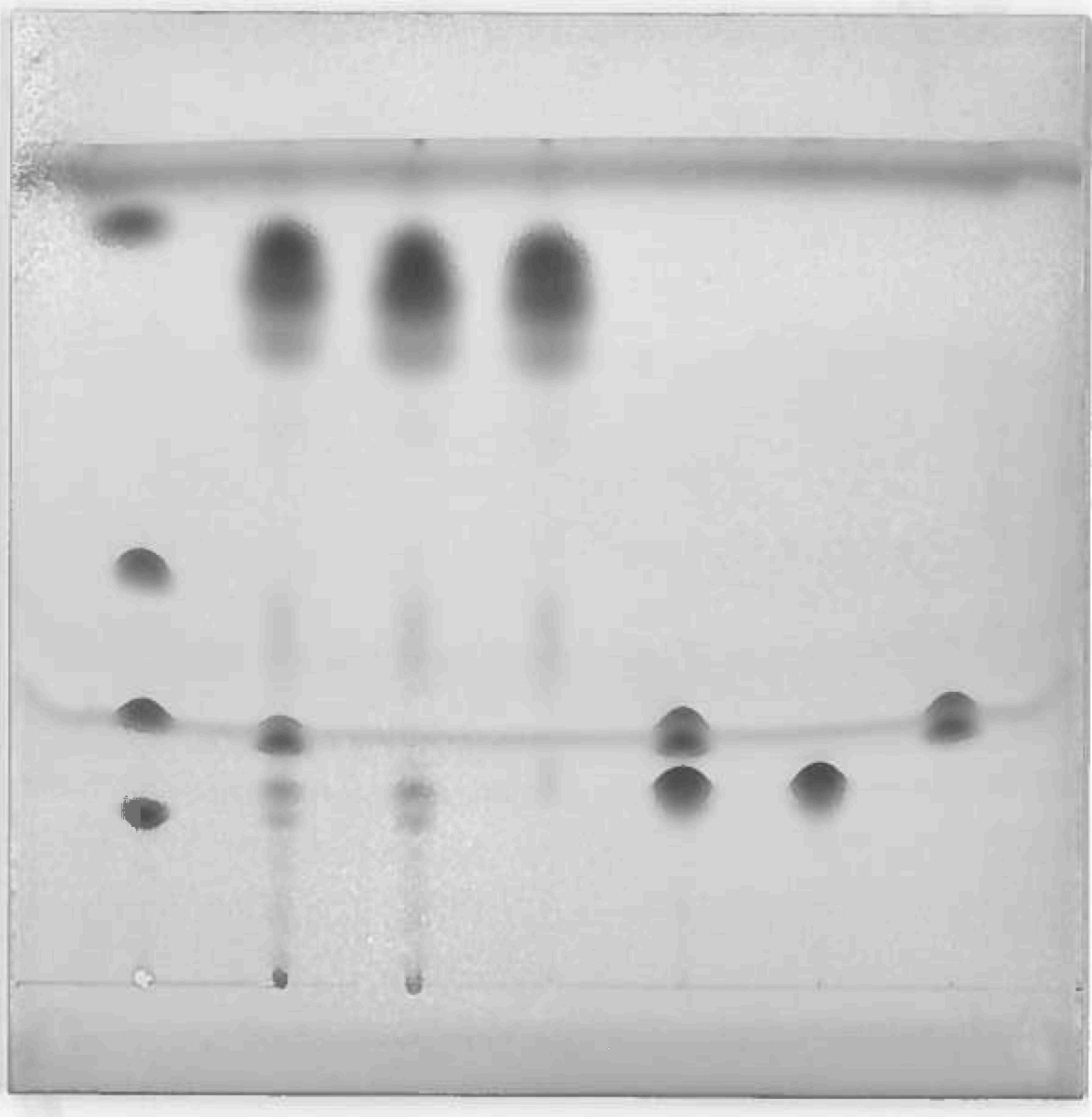

Fig 2 in the manscript. High performance thin layer chromatography of the lipid classes isolated from Calanus oil by SPE. Image captured as a scann on a Xerox WorkCenter 7855i. Columns from left to right: lipid class standard 18-5 A (Nu-Chek Prep, INC. USA) containing from bottom to top lecithin; cholesterol; oleic acid; TAG; and cholesteryl oleate. Oil from C. finmarchicus. Isolated neutral lipids. Isolated wax esters. Hydrolyzed wax esters. Isolated free fatty alcohols and isolated free fatty acids from the hydrolyzed wax ester

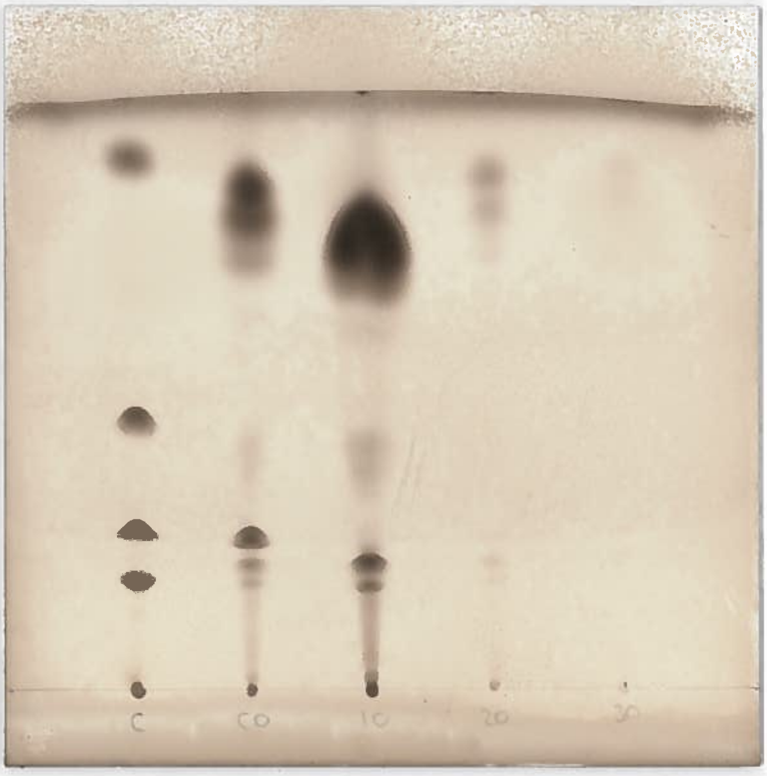


Supportive fig 1: High performance thin layer chromatography of the isolated neutral lipids from 300mg Calanus oil by SPE. Image captured as a scann on a Xerox WorkCenter 7855i. Columns from left to right: lipid class standard 18-5 A (Nu-Chek Prep, INC. USA) containing from bottom to top lecithin; cholesterol; oleic acid; TAG; and cholesteryl oleate. Oil from C. finmarchicus. Neutral lipids (NL) eluted with 10 mL chloroform/isopropanol (2:1 v/v), NL eluted with an additional 10 mL (20 mL in total), NL eluted with a  additional 10 mL (30 mL in total).

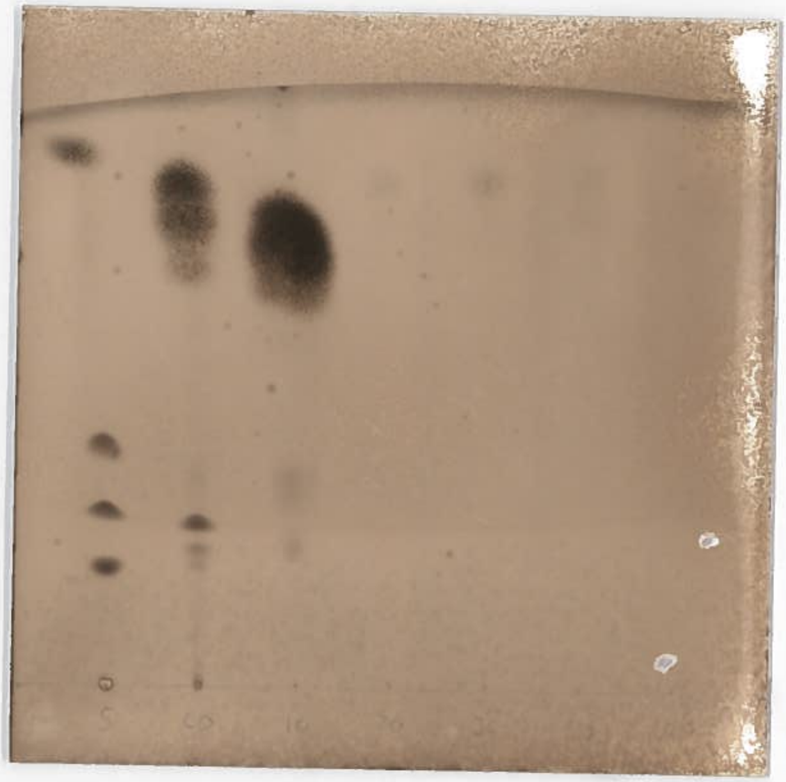

Supportive Fig 2: High performance thin layer chromatography of wax esters isolated by SPE from the neutral lipids from 300mg Calanus oil. Image captured as a scann on a Xerox WorkCenter 7855i. Columns from left to right: lipid class standard 18-5 A (Nu-Chek Prep, INC. USA) containing from bottom to top lecithin; cholesterol; oleic acid; TAG; and cholesteryl oleate. Oil from C. finmarchicus. Wax esters eluted with 10 mL heptane. Wax esters eluted with an additional 10 mL (20 mL in total). Wax esters eluted with an additional 10 mL (30 mL in total). Wax esters eluted with an additional 10 mL (40 mL in total). Wax esters eluted with a final additional 10 mL heptane (50 mL in total).

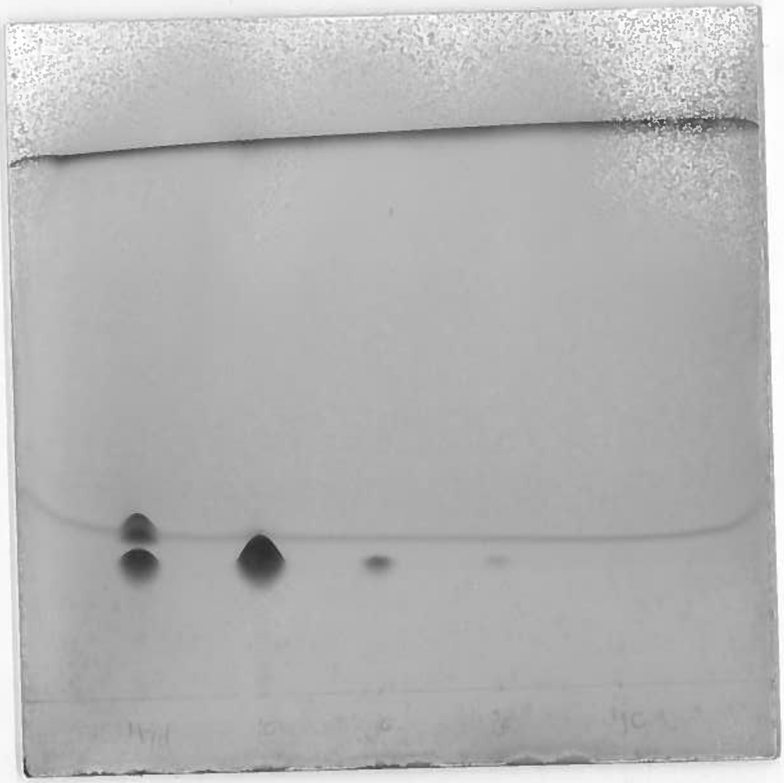

Supportive Fig 3: High performance thin layer chromatography of isolated fatty alcohols from hydrolyzed wax esteres originating from 300 mg Calanus oil. Image captured as a scann on a Xerox WorkCenter 7855i. Columns from left to right: hydrolyzed wax esters. Free fatty alcohols eluted from the hydrolysed wax esters with 10 mL chloroform/isopropanol (2:1 v/v).Free fatty alcohols eluted with an additional 10 mL (20 ml in total). Free fatty alcohols eluted with an additional 10 mL (30 ml in total). Free fatty alcohols eluted with a final additional 10 mL chloroform/isopropanol (2:1 v/v) (40 ml in total).

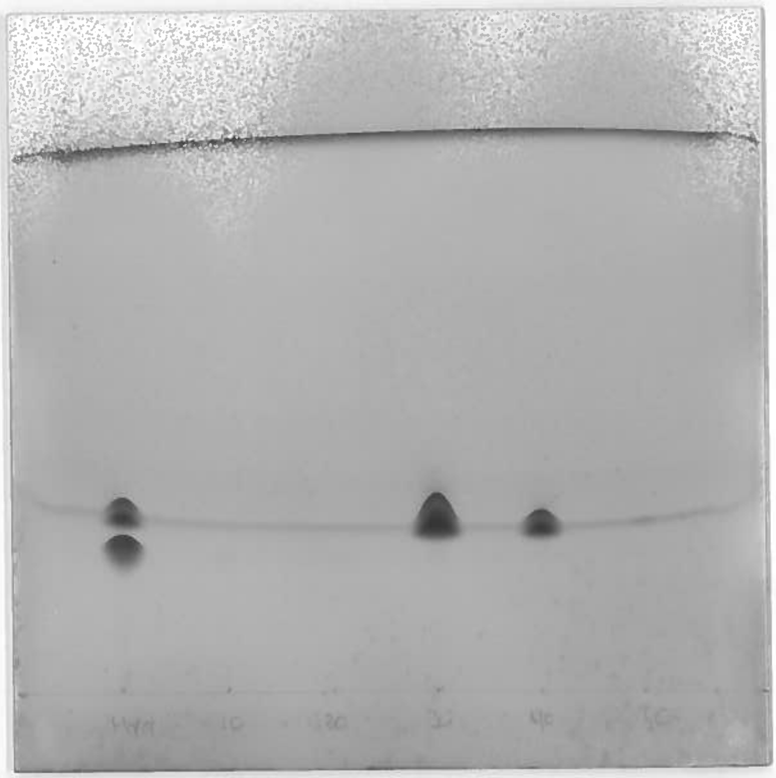

Supportive Fig 4: High performance thin layer chromatography of isolated fatty acids from hydrolyzed wax esteres originating from 300 mg Calanus oil. Image captured as a scann on a Xerox WorkCenter 7855i. Columns from left to right: Hydrolyzed wax esters. Free fatty acids eluted from the hydrolyzed wax esters with 10 mL diethyl ether/acetic acid (98:2 v/v). Free fatty acids eluted with an additional 10 mL (20 mL in total). Free fatty acids eluted with an additional 10 mL (30 mL in total). Free fatty acids eluted with an additional 10 mL (40 mL in total). Free fatty acids eluted with a final additional 10 mL diethyl ether/acetic acid (98:2 v/v) (50 mL in total).
