# Supplementary material for: A simple method to isolate fatty acids and fatty alcohols from wax esters in a wax-ester rich marine oil
Source: PLoS One. 2023 May 12;18(5):e0285751. doi: 10.1371/journal.pone.0285751 (PMC10180661; doi:10.1371/journal.pone.0285751)
Supplement: Attachment — Submitted filename: Response to Reviewers.docx [file pone.0285751.s004.docx]

Dear editor Ramanan,

We would like to thank all reviewers for their constructive feedback. We have gone through each comment and have changed the manuscript accordingly. Regarding the comments from the editorial office:

1. we have changed the files names so that it meets PLOS ONE’s style requirements.
2. We have added two supporting information files (“Supportive figs” and “Supportive table 1”) so that all data that underly the findings we describe in the manuscript are now fully available.
3. We have included a pdf file named “S1_raw_images” that includes all raw Xerox scans from the thin layer chromatography images that we present in the figure and the newly added supplementary data file. Below each image we describe what the image represents and how it has been captured.

Please see below in red our response to each point raised by the reviewers. The indicated line number refer to the manuscript with track changes.

**Reviewer #1:**

The manuscript presents a simplified method to isolate fatty acids and fatty alcohols from a wax ester obtained from a zooplankton rich in monounsaturated fatty acids using HPTLC. The method seems to be really simple and not expensive. The procedures were described step by step and can be useful for other researchers. However, there are some parts that should be revised.

In general, there are several minor typos in the text.

We have gone through the manuscript and have corrected several typos.

Line 107: Include the volume of wax esters in chloroform that resulted in 240 mg

We have included the average weight of wax esters obtained from the three extraction rounds in line 119 and mentioned that they are dissolved in 5mL chloroform.

line 110: it was described that the sample was kept at 90oC/90 min. Usually, an antioxidant uses to be added to avoid oxidative reaction at this step of the analysis.

We did not add an antioxidant during the hydrolyzation step since Calanus oil contains naturally the antioxidant astaxanthin, which gives the oil its red color.

Lines 143-154: include the reference of this part.

We have added on line 177-180 that the composition analyses of the fatty acids and fatty alcohols was done by the company Akvaplan-niva following their inhouse protocol that is based on methods described by J. Folch, M. Lees and Sloane Stanley; J. Biol. Chem., 226, 497 (1957) and Advances in Lipid Methodology - One (edited by W.W. Christie, Oily Press, Ayr), pp. 1-17 (1992).

Line 157: It is important to detail which sub-type of standard was used to identify the fatty acids and fatty alcohols, since the information viable in the company web site has not been enough.

The FAME standards are still available on the 2021-2024 catalogue on the company’s website. <http://www.nu-chekprep.com/CATALOG%202021-2024.pdf>. The fatty alcohol standard GLC 621 is no longer produced as a combination of alcohols, but each alcohol is available individually. The composition of GLC 621 is now given on line 173-177.

What is Akvaplan-niva ?

We have added on line 178-179 that Akvaplan-niva is a Norwegian company based in Tromsø, Norway.

Table 1: It will be expected to have at least a triplicate of the assay. Without any replication it is not possible to obtain an error estimative.

We have now repeated the isolation two more times. The averages of the triplicate values are given in table 1, both in mg and percentage weight of the oil, with standard deviation. The individual values of each isolation are given in supplementary table 1.

It is interesting to suggest what type of lipids correspond to the difference between waxe esters (239 mg) and neutral lipids (266 mg), and also between neutral lipids and calanus oil (300 mg).

We thank the reviewer for her comment. We had touched upon this topic in lines 188-191 but have extended this in lines 191-196.

It is important to better discuss the advantages and limitations of this method compared with others.

We would again like to thank the reviewer for her comment. We have elaborated on the difference between the method we describe here and previously described methods (reference 10, 11, 12 and 13) and the advantages and limitations in the introduction in line 71-77 and in the results and discussion section in line 252-259.

The manuscript is interesting but the lack of replication is not expected in an article about methodology alternatives. In addition, it is also expected a comparison with at least one classical method.
Thus, it is necessary to include replicate and compare with results obtained from other method.

With the 2 extra extractions performed, the average results are presented in table 1, and individual results in supplementary table 1, and the changes we have made to the manuscript we hope we have answered to the reviewer’s constructive feedback.

**Reviewer #2:**

The article describes a method for isolating free fatty acids and free fatty alcohols from wax esters of calanus oil. Although the paper is written very well and the descriptions are clear and sound, I doubt that the level of novelty justifies publication. The steps involved (extraction, saponification, SPE, checked by TLC) are well-described procedures for separating lipid classes and can be found in classic textbooks on lipid analysis (e.g. W.W.Christie…).
We thank the reviewer for their comment. It is indeed true that the methods (SPE and TLC) used here are well-described procedures. We are, however, not aware of any publications describing a simple semi-preparative method for isolation of wax esters present in a wax-ester rich marine oil, and then separating the fatty alcohols and fatty acids constituting these wax esters.

The novelty lies not in the method itself but in the application. As written in the introduction, on line 36-50, there is an increasing need for omega-3 fatty acids, and Calanus oil is a relatively new source of these lipids. In the last couple of years more and more studies on Calanus oil, and its potential health effects, have been done in both animals and humans (line 56-61). To study, in vitro, what part of the oil is responsible for what health effect it is necessary to separate the fatty acids from the fatty alcohols since the cells in our body will not be exposed to intact wax esters but rather to the separate fractions after hydrolyzes in the gut (line 62-69). To our knowledge we are the only lab today that is studying the individual effect of fatty acids and fatty alcohol. But with the growing demand for omega 3 fatty acids and the scientific interest in this oil by other labs worldwide, it can be expected that other labs will perform in vitro studies as well. It will then be of importance that the fatty acid and fatty alcohol fractions will be extracted in the same way to reduce potential confounding effects, through the method we describe here. We therefor do believe this paper justifies publication and we hope the reviewer agrees with us on that.

Furthermore, if this paper is aiming to optimize such a procedure, I would expect some data on method development. The authors merely present data of the (hopefully) optimized method. For method development, it would be essential to know, how the targets were defined (purity, solvent efficiency) and how the optimum was approached.

We again thank the reviewer for their comment. Our main aim was to develop a method that would allow us to obtain the native, non-esterified fatty acids and fatty alcohols from wax esters in Calanus oil. As we show in figure 2, the lipid fractions we have obtained from the hydrolyzed wax esters appear pure. Regarding the optimization of our method, we have now added a supportive data file with TLC analyses from each extraction step described in figure 1, where we qualitatively show the presence of lipids eluted with different volumes of solvent to justify the volume choices in the method described here.

The authors claim that their method works without noticeable oxidation contrary to previous methods. How did the authors check this? I assume, that any oxidized fatty acid or alcohol would be retained in the SPE and thus noted as a reduced recovery.
We do not claim that the method works without noticeable oxidation of fatty acids and fatty alcohols. We agree with the reviewer that if this occurs, oxidized fatty acids and fatty alcohols would be retained in the SPE and result in lower yields. We do, however, claim that we do not oxidize the fatty alcohol to their corresponding fatty acids during the isolation method, which allows us to use the fatty alcohols for further cell cultures. (line 29-33)

As a minor comment, I wonder why the authors did not try to separate FFA from fatty alcohols as a soap before re-acidifying, instead of a SPE, given the fact that both molecular classes have similar polarities. At least this should be discussed.

We have indeed tried to precipitate the FFA as a sodium salt and recover the soluble FaOHs, however we did not get a clear separation as we did with SPE. We have addressed this point now in line 135-138.

**Reviewer #3:** 
The authors describe the preparation of mixtures of fatty acids and fatty alcohol from waxes present in Calanus finmarchicus oil. They propose the use of SPE columns to purify and isolate the waxes, followed by a basic hydrolysis with ethanol and NaOH. The main concerns relate to the methodology used and some of the results described (see specific comments).

Specific comments:

1.-Lines 64-65.- To study the health effects …it is necessary to separate the fatty acids and fatty alcohols …..
Why is it necessary? Provide a reference to support this claim. In any case, is the effect due to the functional class of each compound (alcohol or carboxylic acid) or to the presence of double bonds in the alkyl chain of both classes of compounds?

As mentioned in line 62-69, it is necessary to separate the fatty acids from the fatty alcohols to study if, and how, these individual lipid classes, derived from wax esters, influence human health. In a previous publication, reference 24, (Schots PC, Pedersen AM, Eilertsen K-E, Olsen RL, Larsen TS. Possible Health Effects of a Wax Ester Rich Marine Oil. Frontiers in Pharmacology. 2020;11(961).) we have described the possible health effects of the different fatty acids and fatty alcohols in general that are present in Calanus oil.

2.-Lines 66-67.- To our knowledge … no method … to separate the fatty acids and
fatty alcohols … without the formation of FAMEs.
What is the problem related to the preparation of FAMEs, the separation of the fatty alcohols from FAMEs and, finally, the recovery of fatty acids by hydrolyzing FAMEs?

We thank the reviewer for their comment. We have now explained on line 255-259 that the hydrolyzes of the wax esters led to a loss of material (as determined by weight). An additional hydrolyzes of the FAMES would further reduce the yield of the fatty acids.

3.- Lines 80-82.- … Phosphoric acid 99,99%, Potassium bicarbonate …..Toluene

Chemical names should be written in lowercase unless they are located after a period.

We thank the reviewer and have changed the manuscript accordingly.

4.- Lines 107-108.- …wax esters …. dissolved in …. 1 M ethanolic NaOH to a concentration of 5 mg/mL…
There is an excess of ethanol related to the amount of water present (needed for the hydrolysis), do fatty ethyl esters not appear during this reaction?
We believe they do not since this would have been visible on the TLC (figure 2)

5.- Various lines ..... minutes...hours ….
Time units should be indicated according IS rules: min, h…
We thank the reviewer and have changed the manuscript accordingly.

6.- Lines 185-187.- free fatty acids band appear to consist of two bands is probably due to different degrees of unsaturation….
The different chain length of each acid can also lead to this situation. Please add this to the manuscript.

We thank the reviewer and have changed the manuscript accordingly (line218).

7.- Lines 223-224.- Table 2
How was the initial content of fatty acids and fatty alcohols in the crude wax determined?
How do the authors explain the differences between SFA, MUFA and PUFA for the fatty acids present in the wax and free fatty acids isolated after the SPE process and further saponification?
How do the authors determine the relative content from fatty acids and fatty alcohols as a whole? Did they consider the different response factors of each in the GC?
What is the fatty acid indicated (0.8 relative content) in the fatty alcohol section of the table?

The fatty acid and fatty alcohol content in the wax esters and isolated lipid fractions was determined by the company Akvaplan-niva via an inhouse protocol as explained on line 161-180.

Table 2 give values in percentage. The values of the different fatty acids in the isolated fatty acid fraction are about twice as high as those in the wax ester sample. This is because in the wax ester sample only half of the sample consists of fatty acids (48,6%), and the other half are fatty alcohols (45,4%).

The fatty alcohol that is present with a relative content of 0,8 in the isolated fatty acid fraction is 22:1 n-11 (docosenol). Minor amounts of a few fatty acids are apparently detected in the fatty alcohol fraction, 1,8% 20:1 n-7 and 3,5% 18-3 n-3 and 2,6% 20:3 n-6.

8.- Figure 1
Did all SPE used contain the same chromatographic phase as described in line 88 (5 g Mega Bond Elute aminopropyl)?

Yes, the same column (5 g Mega Bond Elute aminopropyl) was used for all different SPE steps in this procedure.
