# Supplementary material for: A simple method to isolate fatty acids and fatty alcohols from wax esters in a wax-ester rich marine oil
Source: PLoS One. 2023 May 12;18(5):e0285751. doi: 10.1371/journal.pone.0285751 (PMC10180661; doi:10.1371/journal.pone.0285751)
Supplement: Attachment — Submitted filename: comments.docx [file pone.0285751.s005.docx]

This revised paper has been improved. However, I still have a concern (see specific comments ) about the current paper before recommending its publication.

**Specific comments**

1. **Line 138 …..n in our set-up using NaOH as a salt.**

NaOH cannot be defined as a salt from a chemical point of view. Of course, it can modify the ionic strength of a solution, but as a base. Please amend this sentence.

**2.- Table 2**

The authors do not explain the differences between SFA, MUFA and PUFA for the fatty acids present in the wax and the free fatty acids isolated after the SPE process and further saponification. According to the percentages indicated in Table 2, the percentages referring to 90.2 % of the total FFA isolated should be 26.35, 21.5 and 42.31 for SFA, MUFA and PUFA, respectively considering the initial percentages of fatty acids found in the waxes. However, the percentages found are 32.2, 22.5 and 35.5, which show an increase in SFA and a decrease in MUFA and, even more, in PUFA. In my opinion, this clearly indicates a higher loss of unsaturated versus saturated fatty acid during the sample processing. The authors should clearly explain this in the manuscript.
