# Supplementary material for: A simple method to isolate fatty acids and fatty alcohols from wax esters in a wax-ester rich marine oil
Source: PLoS One. 2023 May 12;18(5):e0285751. doi: 10.1371/journal.pone.0285751 (PMC10180661; doi:10.1371/journal.pone.0285751)
Supplement: Attachment — Submitted filename: Response to reviewers.docx [file pone.0285751.s006.docx]

Response to reviewers

We would like to thank all reviewers again for their constructive feedback and have changed the manuscript accordingly.

**Comments to the editor:**

1. We saw two spelling mistakes in the names of the last two authors of reference 16 in the reference list and have changed that accordingly. The changes are highlighted in yellow in the manuscript with track changes.
2. Upon request by the journal, we uploaded a new supplementary figure 3 and 4 which now includes a legend (a and b) explaining which band represents the fatty alcohols and fatty acids in the hydrolyzed wax esters in lane 1.
3. Also upon request by the journal, we deleted the funding statement, regarding the publication fee, at the end of the manuscript. This sentence will be moved to the Funding Statement in the online submission form and is now, upon request by the journal, included in the cover letter.

Please see below in red our response to each point raised by the reviewers. The indicated line number refer to the manuscript with track changes. In addition, some minor linguistic changes have been made on line 44, 69, 73, 109, 171, 173 and 179.

**Reviewer #1:**

Line 60: making:

We have changed the sentence on line 60, making it one sentence instead of two.

Line 122: It is important to include the authors answer in the text. For example: “No antioxidants were added during the wax esters hydrolysis because Calanus oil contains naturally the antioxidant astaxanthin”.

We have added the sentence as suggested by the reviewer on line 122-123

**Reviewer #2:**

Reviewer #2: In my view, the authors have clarified all points satisfactorily. There is just one point that popped in to my eyes: In the description of figures, it should read."...from bottom to top.." - not top to bottom (all figures and supportive figures).

This is indeed correct, and we thank the reviewer for pointing this out. We have changed the figure legend as the reviewer mentions in the figure text of the raw images. In the figure text in the manuscript and the supplementary data file the different lipid classes are identified with a letter and we therefore did not add the phrase “from bottom to top”.

**Reviewer #3**

**Line 138 …..n in our set-up using NaOH as a salt.**

NaOH cannot be defined as a salt from a chemical point of view. Of course, it can modify the ionic strength of a solution, but as a base. Please amend this sentence.

This is indeed correct, and we have changed the sentence on line 136-137 accordingly.

**2.- Table 2**

The authors do not explain the differences between SFA, MUFA and PUFA for the fatty acids present in the wax and the free fatty acids isolated after the SPE process and further saponification. According to the percentages indicated in Table 2, the percentages referring to 90.2 % of the total FFA isolated should be 26.35, 21.5 and 42.31 for SFA, MUFA and PUFA, respectively considering the initial percentages of fatty acids found in the waxes. However, the percentages found are 32.2, 22.5 and 35.5, which show an increase in SFA and a decrease in MUFA and, even more, in PUFA. In my opinion, this clearly indicates a higher loss of unsaturated versus saturated fatty acid during the sample processing. The authors should clearly explain this in the manuscript.

Also this is correct and we thank the reviewer for pointing this out. We have added a part on line 243-248 to explain this.
